# Supplementary material for: bacLIFE: a user-friendly computational workflow for genome analysis and prediction of lifestyle-associated genes in bacteria
Source: Nat Commun. 2024 Mar 7;15:2072. doi: 10.1038/s41467-024-46302-y (PMC10920822; doi:10.1038/s41467-024-46302-y)
Supplement: Supplementary file 1 — Supplementary Information [file 41467_2024_46302_MOESM1_ESM.pdf]

Supplementary Figure 1

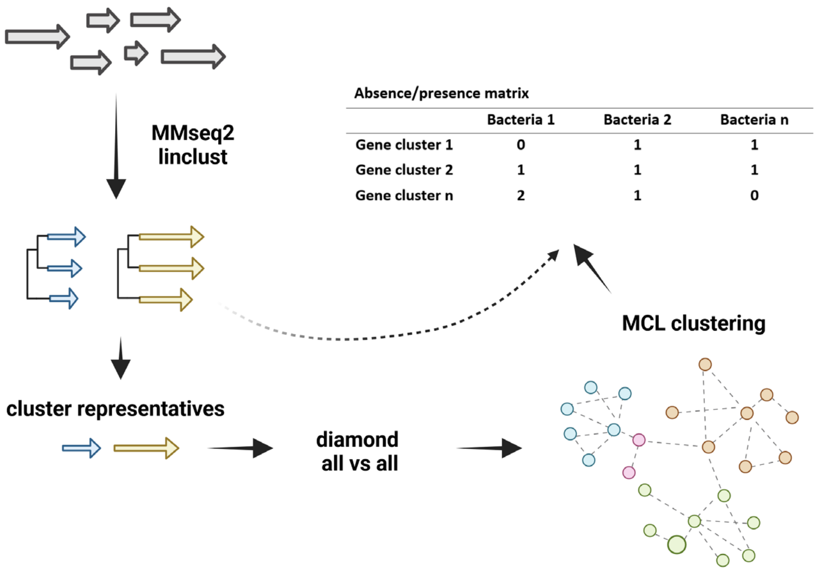

Supplementary Figure 1. Clustering framework performed within bacLIFE to generate functional gene clusters. Genes are merged into a single fasta file which is used as input for MMseq2[40] linclust clustering at the 90% similarity and 80% minimum coverage. Representatives of each MMseq2 cluster are used as input for a diamond alignment of all vs all genes. Alignment statistics are parsed into a network format using the bit score as edge weight. This network is used as input for the Markov clustering[85] to generate clusters based on graph theory. Results from MCL and MMseq2 are combined to generate a gene cluster absence/presence matrix. Created with BioRender.com

Supplementary Figure 2

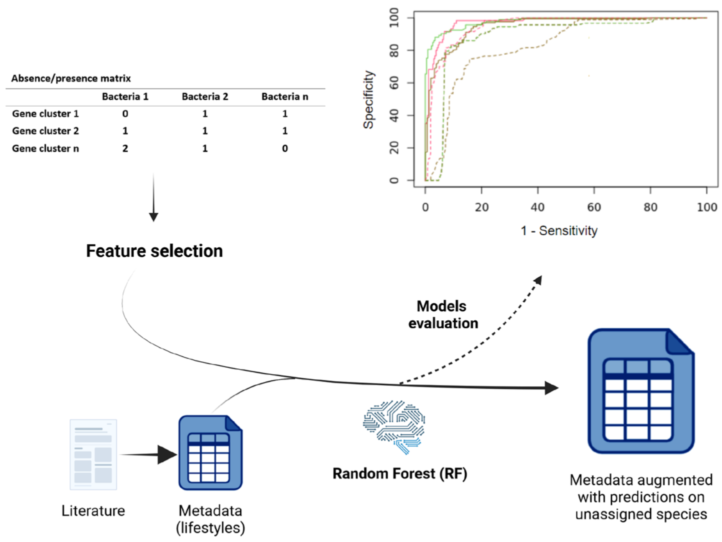

Supplementary Figure 2. Overview of the lifestyle prediction module. Gene absence/presence matrix is used as input. First, the number of variables is reduced with a chi-square based feature selection. Lifestyle metadata is collected from literature and its predictability is tested with machine learning models such as random forest and nearest neighbor. Model evaluation was performed looking into Receiver Operating Characteristic curves (ROC plots) and area under the curve (AUC) values of predictions made in a test set following a 5-fold cross validation scheme. After model evaluation, if model predictability is accurate, the trained model is used to augment the metadata and predict the lifestyle of unassigned species or unknown lifestyle bacteria. Created with BioRender.com

Supplementary Figure 3

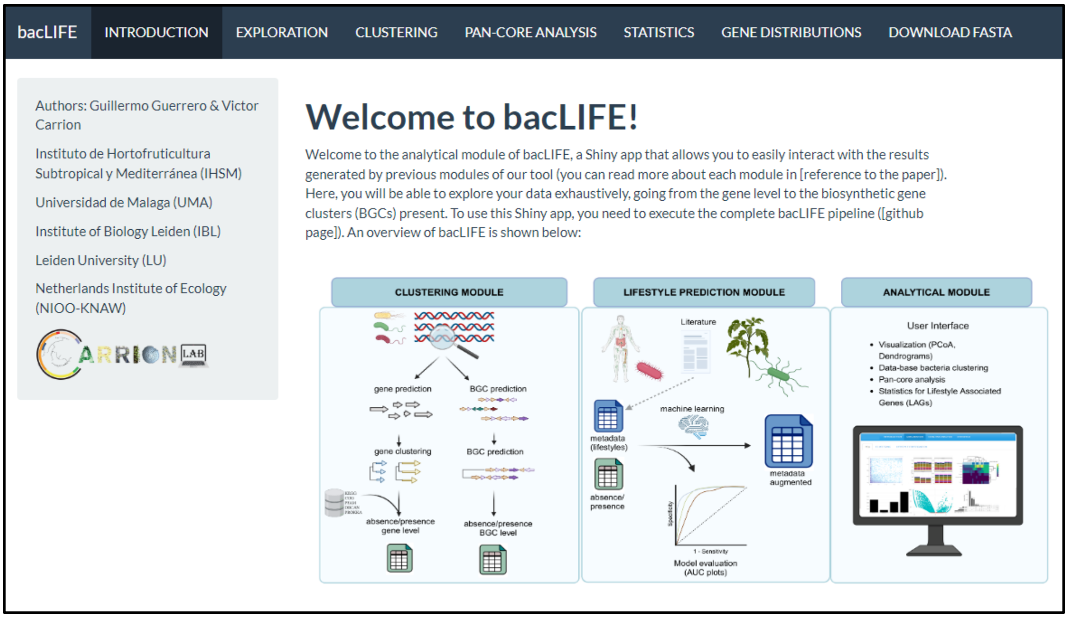

Supplementary Figure 3. Unveiling Gene Cluster Distributions through bacLIFE app. Explore and analyze gene cluster distributions using the interactive user interface, leveraging the output of the clustering module. For additional details, visit our GitHub repository at https://github.com/Carrion-lab/bacLIFE. bacLIFE flowchart shown in the app was created with BioRender.com

Supplementary Figure 4

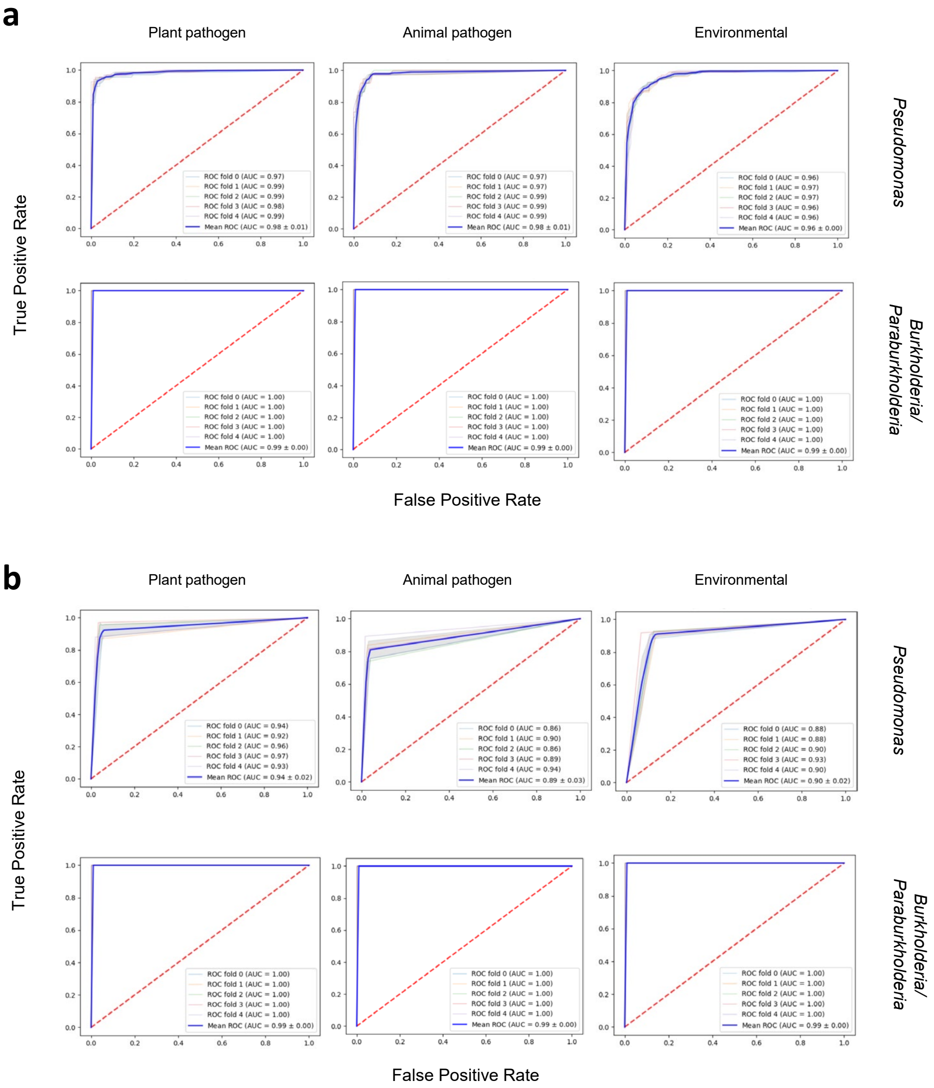

Supplementary Figure 4. Machine learning performance for lifestyle prediction. a, Receiver Operating Characteristic (ROC) curves of the random forest and **b,** nearest neighbor predictions for the three lifestyles defined in the *Pseudomonas* and *Burkholderia/Paraburkholderia* datasets. Area Under the Curve (AUC) values for each lifestyle prediction of the two models are calculated as the mean AUC of the 5-fold cross-validation individual AUCs.

Supplementary Figure 5

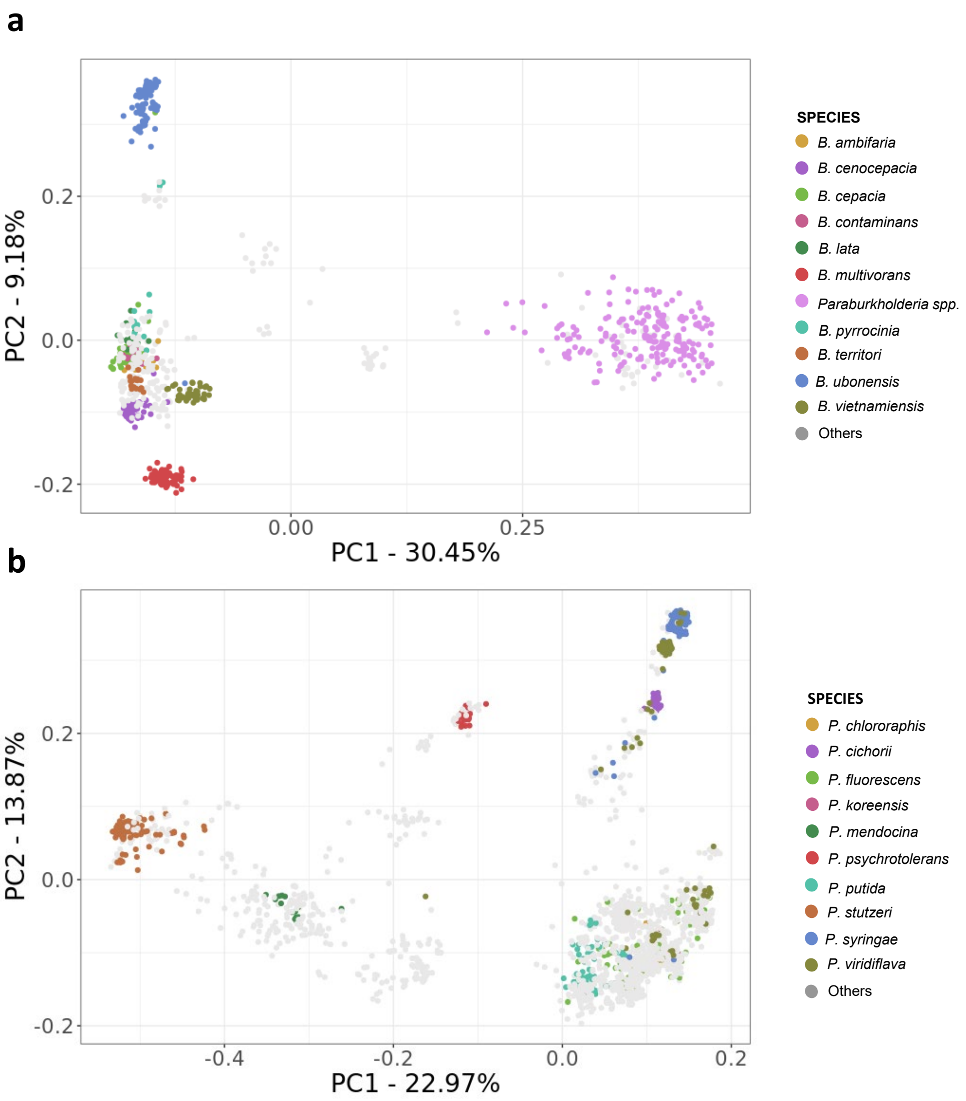

Supplementary Figure 5. A systematic analysis of the *Burkholderia/Paraburkholderia* and *Pseudomonas spp.* species. a and **b,** Principal Coordinate Analysis (PCoA) plots based on the dice dissimilarity calculated using the absence/presence matrix output by the bacLIFE clustering module for *Burkholderia/Paraburkholderia* and *Pseudomonas spp*., respectively. Each point in the plot represents a genome, and the color indicates the species.

Supplementary Figure 6

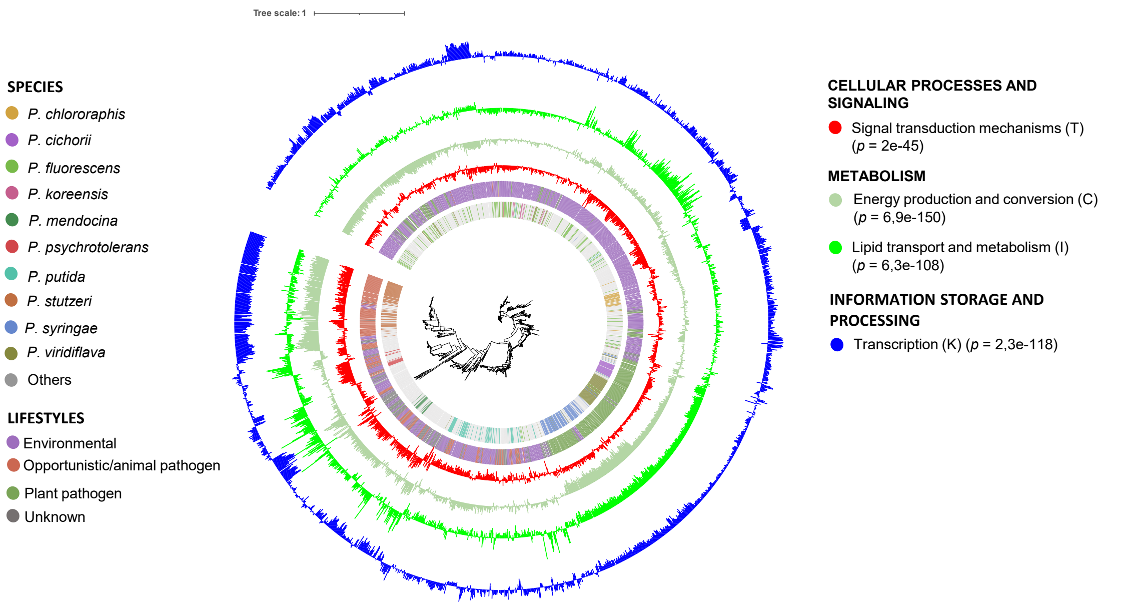

Supplementary Figure 6. **Functional categories of COGs (Clusters of Orthologous Groups) and their correlation with the predicted lifestyles in the *Pseudomonas* genera.** Phylogenetic analysis of the *Pseudomonas* genera (n = 2050) based on concatenated alignment (11001 positions) 77 ubiquitously conserved proteins identified with PhyloPhlAn 3.0[102] and visualized/annotated using iTOL[106]. The first two inner colour coded rings are depicting the *Pseudomonas* species used in this study and their associated lifestyle. The phylogenetic tree is also decorated with three groups of bar plots representing the abundance of the most significant (Kruskal-Wallis, P <0.05) genes associated with some selected COG functional categories (from the inside to outside): The red bar plot is representing the Cellular Processes and Signaling category, including the subcategories of: Signal transduction mechanism (T). The green bar plots are depicting the Metabolism category, including the subcategories of: Energy production and conversion (C) and Lipid transport and metabolism (I). The blue bar plots are representing the Information storage and processing category, including the subcategories of Transcription (K). The bar plots can display negative values because the zero point on the plot represents the mean percentage of that COG category in the entire dataset. Negative values indicate a proportion below the genus average, while positive values signify a proportion above the genus average. Mean proportion of the *Pseudomonas* COG (Tatusov et al. 2000) categories (from inside to outside: T 6%, C 4.97%, I 5%, K 7.53%).

Supplementary Figure 7

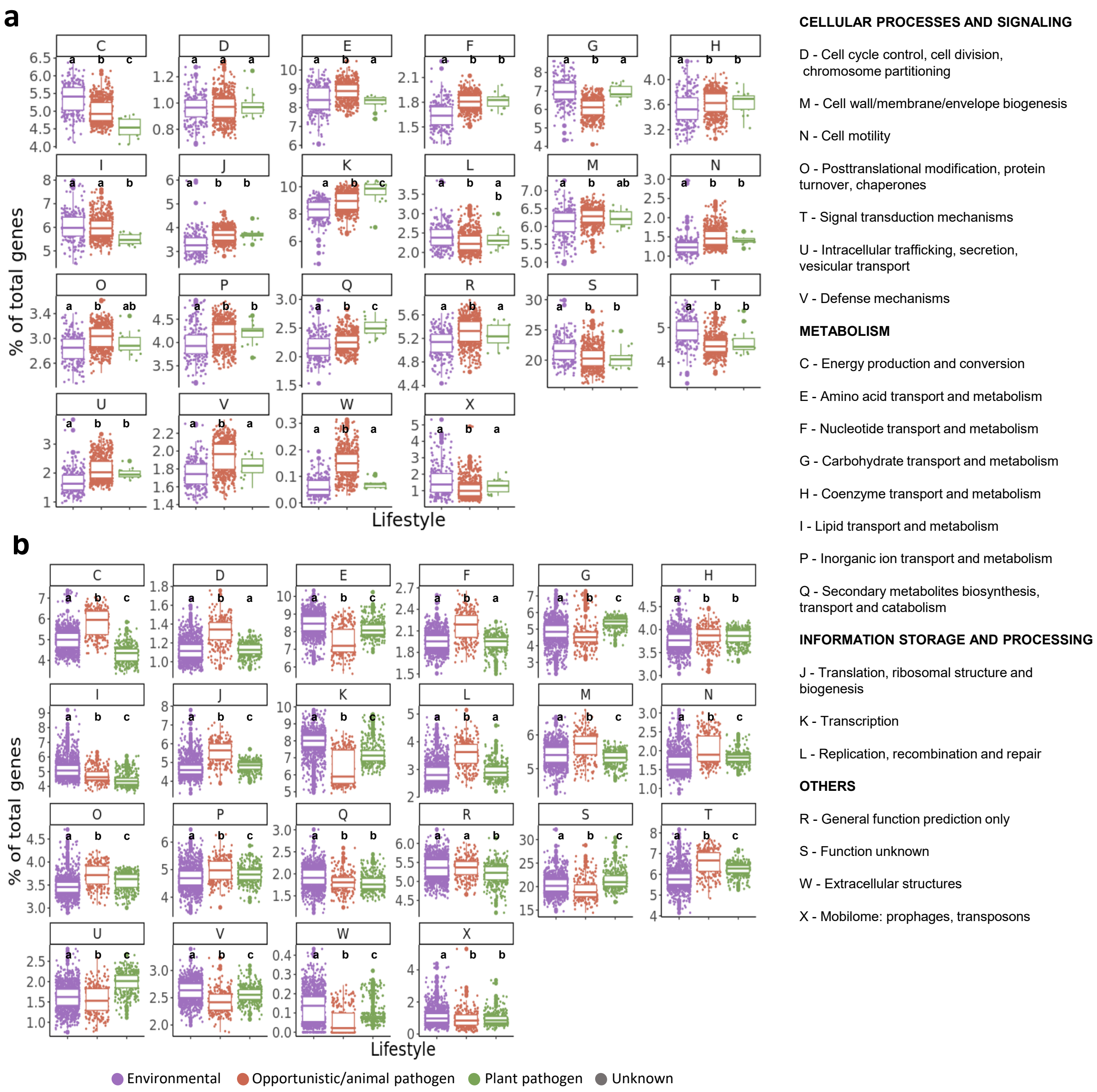

Supplementary Figure 7. Relative abundance of Clusters of Orthologous Groups (COG) functional categories distribution among the three different lifestyles. Boxplots of the relative abundance of COG[45] categories in the three lifestyles for the **a,** *Burkholderia/Paraburkholderia* and **b,** *Pseudomonas* genera. Each point represents one genome and values in the y axis indicate the proportion of genes that are associated with a specific COG category. Using Krustal-Wallis statistical test we found that all COG categories show significant differences among these 3 lifestyles in both genera, except D (Cell cycle control, cell division, chromosome partitioning) in *Burkholderia*/*Paraburkholderia*. The significance levels in every COG category are shown above every box. Each COG type has been abbreviated D: cell cycle control, cell division, and chromosome partitioning, M: cell wall/membrane/envelope biogenesis, N: cell motility, O: post-translational modification, protein turnover, and chaperones, T: signal transduction mechanisms, U: intracellular trafficking, secretion, and vesicular transport, V: defense mechanisms, W: extracellular structures, Y: nuclear structure, B: chromatin structure and dynamics, J: translation, ribosomal structure, and biogenesis, K: Transcription, L: replication, recombination, and repair, C: energy production and conversion, E: amino acid transport and metabolism, F: nucleotide transport and metabolism, G: carbohydrate transport and metabolism, H: coenzyme transport and metabolism, I: lipid transport and metabolism, P: inorganic ion transport and metabolism, Q: secondary metabolites biosynthesis, transport, and catabolism, R: general function prediction only and S: function unknown.

Supplementary Figure 8

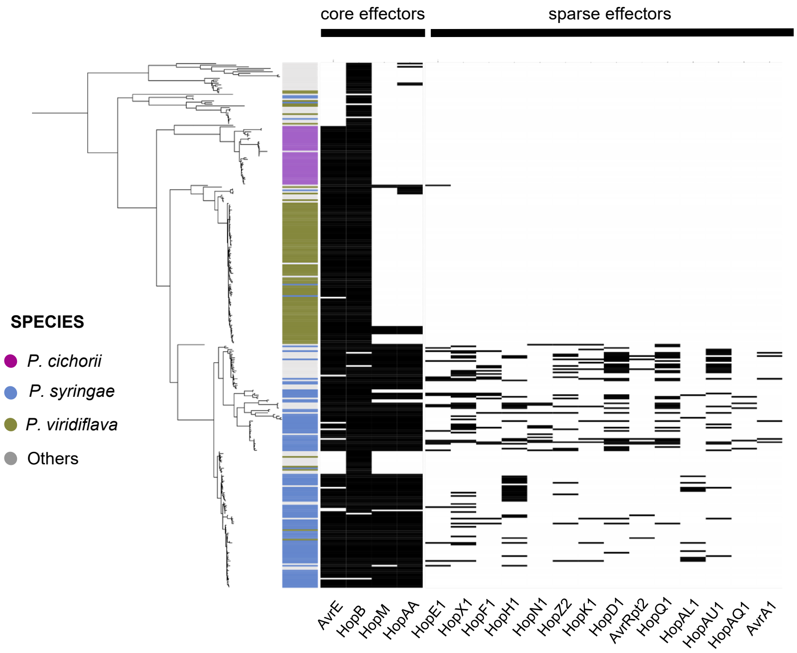

Supplementary Figure 8. **Distribution of the well-studied Type III Secretion System Effectors from the *P. syringae* group.** Phylogenetic analysis of the *Pseudomonas* genera (n = 332) based on concatenated alignment (11001 amino-acid positions) 77 ubiquitously conserved proteins identified with PhyloPhlAn 3.0[102] and visualized/annotated using iTOL[106]. The phylogenetic tree is coupled with a colored strip highlighting the *P. syringae*, *P. viridiflava* and *P. cichori* species, typically associated with plant pathogenic lifestyle. In order to evaluate the efficacy of bacLIFE gene clusters, 26 well-known effectors were mapped to bacLIFE to check their distribution and confirm what is in literature. Most *P. syringae* bacteria are known to possess the T3SS effectors AvrE, HopB, HopM, and HopAA, while the remaining effectors are sparsely distributed within this species, as confirmed by the bacLIFE gene distribution data.

Supplementary Figure 9

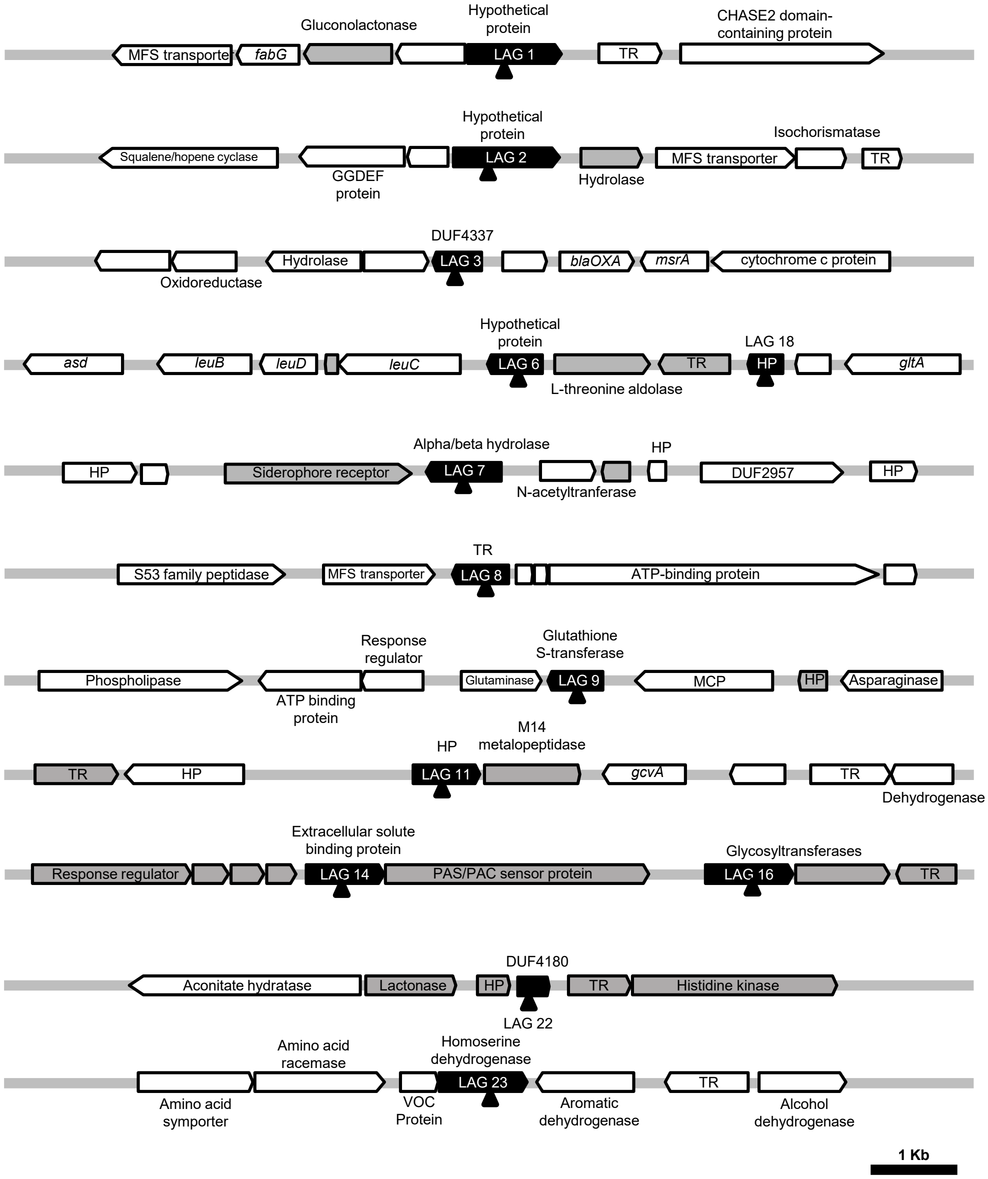

Supplementary Figure 9. **Schematic map of the genetic context of the 13 plant pathogen lifestyle associated genes (LAGs) selected in *B. plantarii* DSM 9509 using bacLIFE.** Genes selected for mutagenesis are indicated in black and genes significantly associated with plant pathogen lifestyle are indicated in gray. Allocation of the insertion using the pSHAFT2 vector is indicated with an arrow. HP, hypothetical protein; TR, transcriptional regulator; MCP, methyl accepting chemotaxis protein; MFS, major facilitator superfamily. While the bacLIFE app enables the identification of consecutive LAG regions using positional information, it relies solely on this data. Further investigation into these regions, including transcription direction, was conducted using other genome visualization tools such as IGV

Supplementary Figure 10

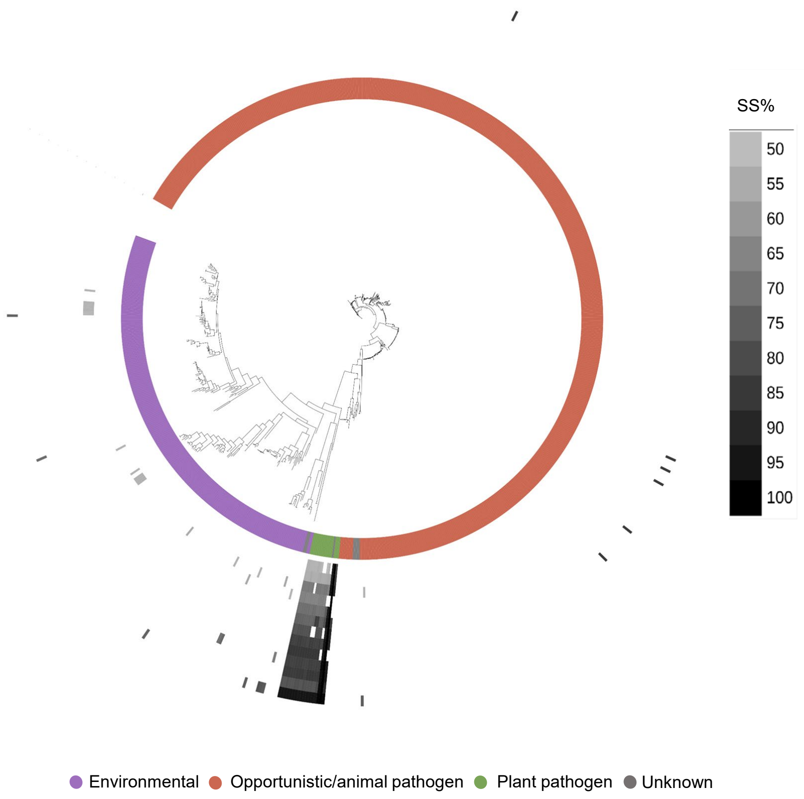

Supplementary Figure 10. **Blastp analysis confirms the results of bacLIFE about the exclusivity of the 13 plant pathogen Lifestyle Associated Genes (LAGs) selected in the *Burkholderia/Paraburkholderia* genera.** Phylogenetic analysis of the *Burkholderia/Paraburkholderia* genera (n = 845) based on concatenated alignment (2146 amino-acid positions) 54 ubiquitously conserved proteins identified with PhyloPhlAn 3.0[102] and visualized/annotated using iTOL[106]. The first inner colour coded ring is depicting the *Burkholderia/Paraburkholderia* associated lifestyle. The heatmap shows the sequence similarity (SS) of the blastp analysis performed using as query the 15 phytopathogenic LAGs chosen for experimental validation (from inside to outside: LAGs 18, 1, 2, 8, 6, 9, 14, 16, 23, 7, 22, 11, 3).

Supplementary Figure 11

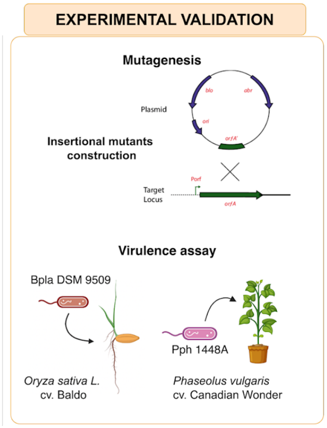

Supplementary Figure 11. **bacLIFE experimental validation workflow.** A selection of predicted lifestyle associated genes (LAGs) by bacLIFE were selected for mutagenesis. Virulence assays were performed using *Burkholderia plantarii* DSM 9509 (Bpla DSM 9509) and *Pseudomonas syringae* pv. phaseolica 1448A (Pph 1448A) in rice (*Oryza sativa* L. cv. Baldo) and bean plants (*Phaseolus vulgaris* cv. Canadian Wonder), respectively. Created with BioRender.com

Supplementary Figure 12

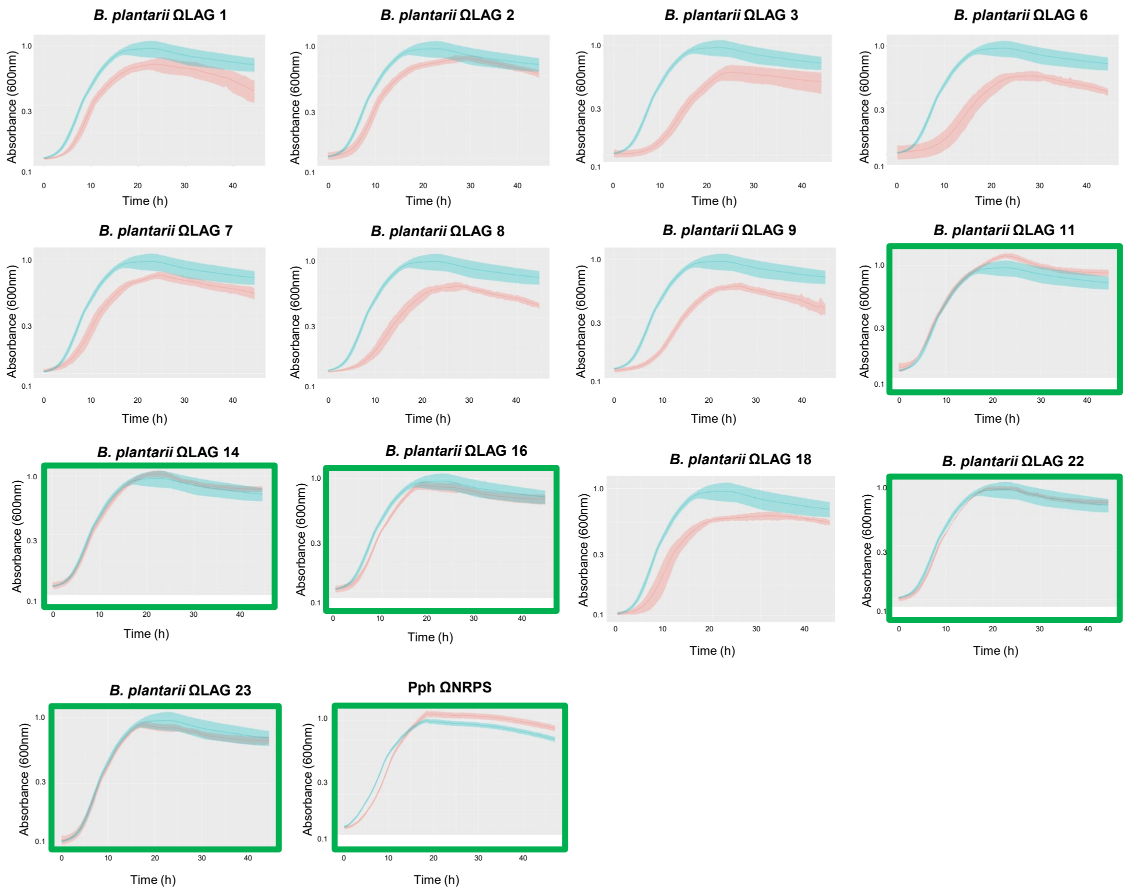

Supplementary Figure 12. **Comparison of growth of wild type and single deletion mutant strains.** Comparison of bacterial growth curves between *B. plantarii* DSM 9509 and *P. syringae* pv. phaseolica 1448A wild-type strains (blue) and the 15 *Burkholderia* LAGs and NRPS mutants (red) constructed in this study plotted in log 10 scale. All strains were grown in LB media during 48 hours. Each curve was constructed using three biological replicates with twelve technical replicates each, with standard deviation calculated accordingly and represented as error bars. Plots squared in green are highlighting the mutants not affected in growth and therefore selected for further experimental validation.

Supplementary Figure 13

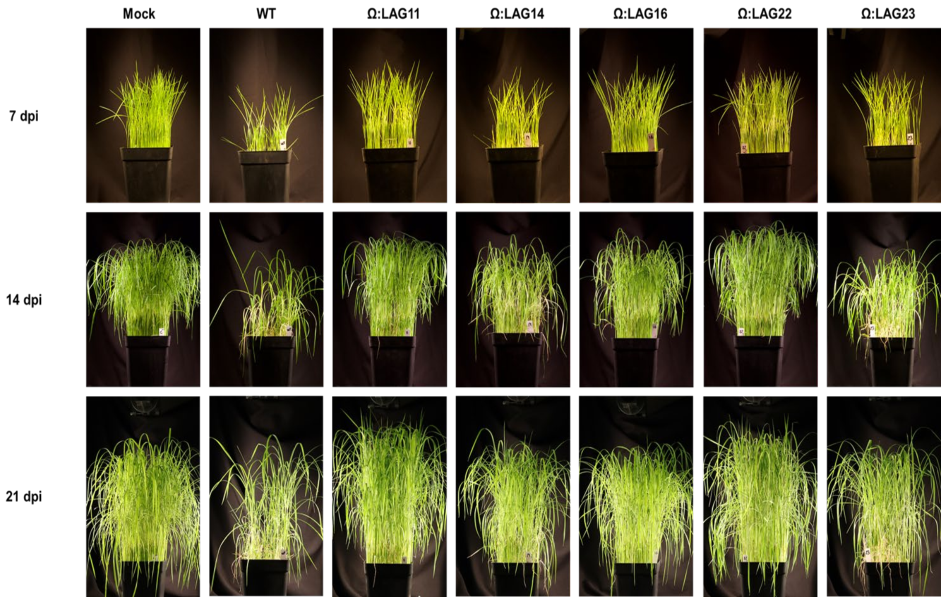

Supplementary Figure 13. **Virulence assay of *B. plantarii* and five LAGs mutants in soil.** Bacterial suspensions adjusted to an OD_{600nm} of 0.5 ($1x10^8$ CFU gr $soil^{-1}$) were inoculated in the soil before sowing the pregerminated seeds. The results showed that the mutants and the non-inoculated rice plants had a similar phenotype.

Supplementary Figure 14

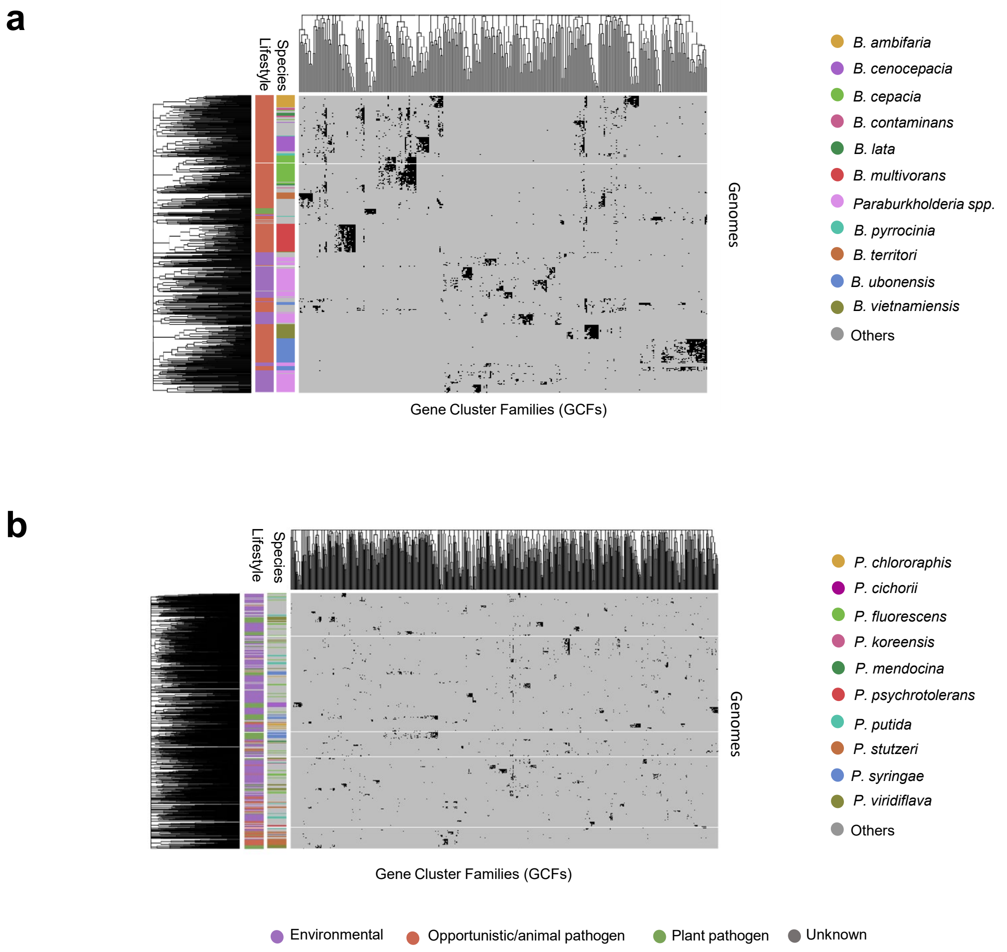

Supplementary Figure 14. **Biosynthetic Gene Clusters (BGCs) distribution in *Burkholderia/Paraburkholderia* and *Pseudomonas* genera.** BiG-SCAPE[42] was used to cluster the BGCs and reduce the complexity of the analysis in a total of 1279 and 856 GCFs (Gene Cluster Families) in *Pseudomonas* and *Burkholderia/Paraburkholderia* respectively. The distribution of all GCFs is shown as a clustered heatmap for the *Burkholderia/Paraburkholderia* **a,** and *Pseudomonas* **b,** genera. Heatmap clustering is performed by the package heatmap.2 based on a binary distance matrix calculated with the input data given for the function. Colored strips indicate the lifestyle (left) and the species annotations (right) in the dataset. The BGCs were mainly grouped into Gene Cluster Families (GCFs) based on their species and lifestyle association. Fisher's exact test ($P < 0.01$ and $> 15\%$ group presence) was used to find plant pathogen associated BGCs (Table S6 and S7). Light gray indicates the absence of GCFs, whereas black indicates the presence of GCFs.

Supplementary Figure 15

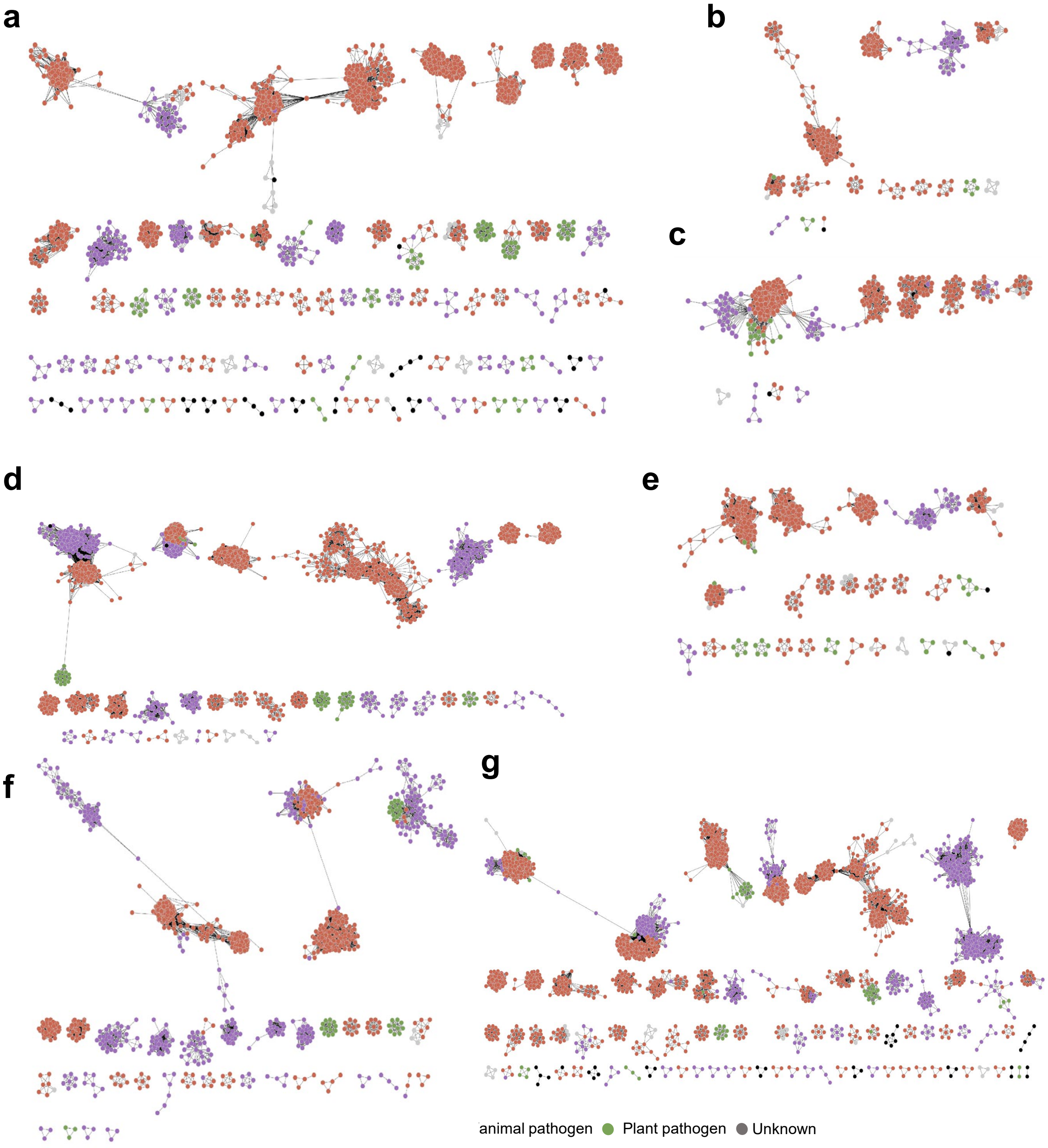

Supplementary Figure 15. **Diversity and distribution of biosynthetic gene clusters of *Burkholderia/Paraburkholderia.*** Biosynthetic Gene Clusters (BGC) sequence similarity networks constructed with BiG-SCAPE[42] using a threshold of 0.3 for **a,** NRPSs, **b,** PKS-NRPS, **c,** PKSI, **d,** terpenes, **e,** PKS others, **f,** RiPPs and **g,** others. Node colors represent the lifestyle where that BGC was found. Nodes with less than three connections were removed.

Supplementary Figure 16

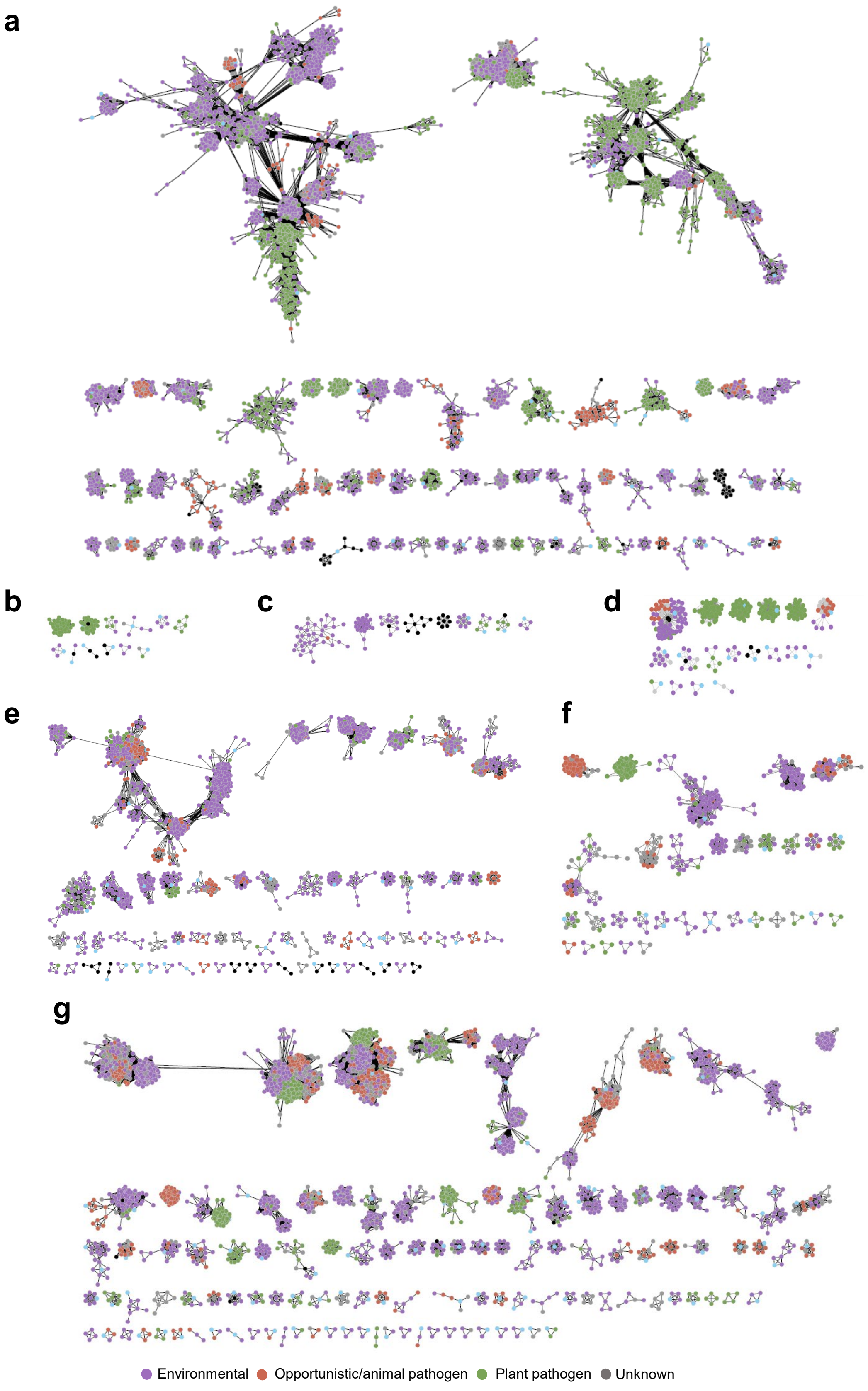

Supplementary Figure 16. **Diversity and distribution of biosynthetic gene clusters of *Pseudomonas.*** Biosynthetic Gene Clusters (BGC) sequence similarity networks constructed with BiG-SCAPE[42] using a threshold of 0.3 for **a,** NRPSs, **b,** PKS-NRPS, **c,** PKSI, **d,** PKS others, **e,** RiPPs, **f,** terpenes and **g,** others. Node colors represent the lifestyle where that BGC was found. Nodes with less than three connections were removed.

Supplementary Figure 17

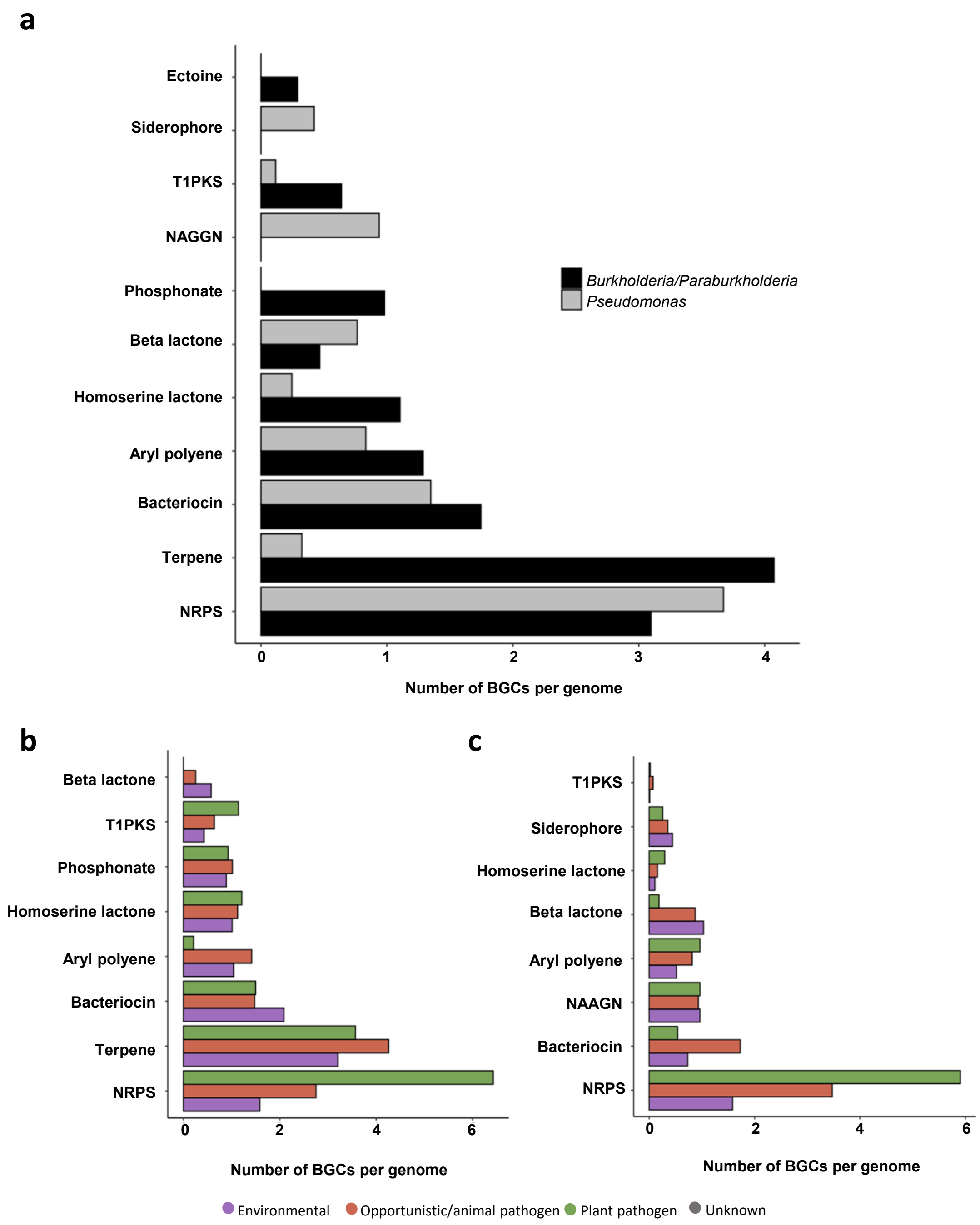

Supplementary Figure 17. **Genome relative abundance of functional categories of Biosynthetic Gene Clusters (BGCs) in *Burkholderia/Paraburkholderia* and *Pseudomonas* datasets**. **a,** The bar plot illustrates the average number of BGCs per genome for the top 10 more abundant functional type categories of each dataset, *Burkholderia/Paraburkholderia* and *Pseudomonas*, as extracted from BiG-SCAPE output. The black bars indicate *Burkholderia/Paraburkholderia*, while the grey bars represent *Pseudomonas* average BGCs per genome. Y-axis abbreviations: T1PKS (Type I Polyketide Synthase) , NAGGN (N-AcetylGlutaminylGlutamine Amide Synthase) and NRPS (Non-Ribosomal Peptide Synthase). Similar bar plot but grouped and colored by lifestyle is shown for *Burkholderia/Paraburkholderia* **b,** and *Pseudomonas* **c,** respectively. In both datasets the average number of NRPSs per genome is higher in the plant pathogen lifestyle.

Supplementary Figure 18

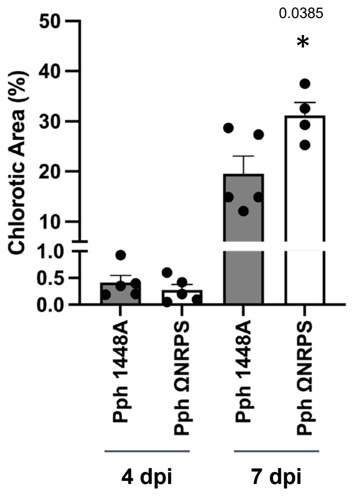

Supplementary Figure 18. **Complementary analyses of *P. syringae* pv. phaseolica 1448A (Pph 1448A) and Pph NRPS mutant virulence in bean leaves (*Phaseolus vulgaris* cv. Canadian Wonder).** Quantification of chlorotic areas as a percentage of chlorotic lesion per infiltrated area in the inoculated leaves shown in Fig. 7e. Error bars correspond to the standard deviation of five different leaves from two independent experiments. Asterisks indicate significantly differences between wild-type and mutant strains using Student's *t* test ($P \leq 0.05$).

Supplementary Figure 19

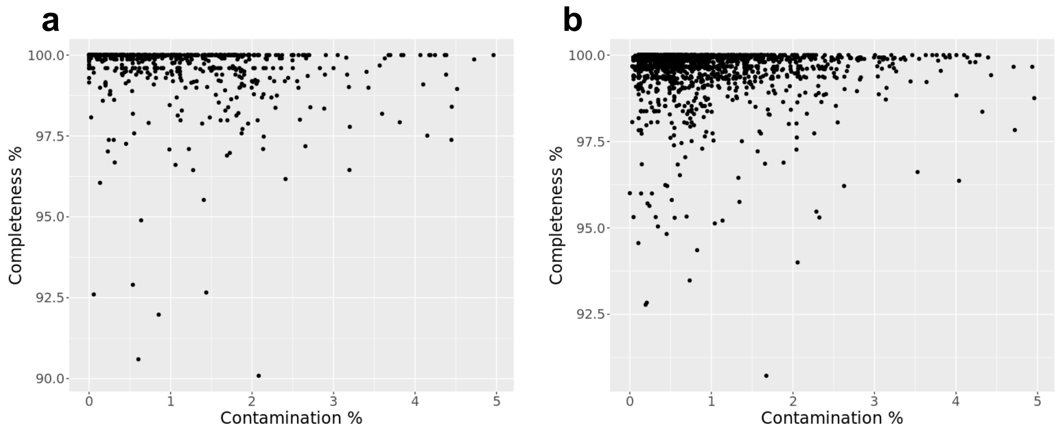

Supplementary Figure 19. CheckM contamination and completeness of the genomes used in this study. For each genome belonging to *Burkholderia/Paraburkholderia* **a,** and *Pseudomonas* **b,** contamination and completeness are plotted as a scatter plot with each point representing one genome

Supplementary Figure 20

***Burkholderia/Paraburkholderia* dataset**

a

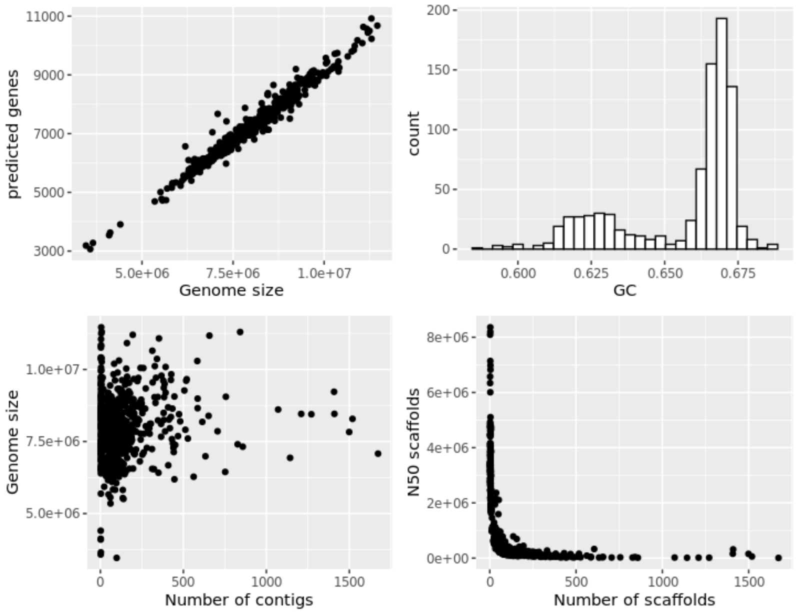

***Pseudomonas* dataset**

b

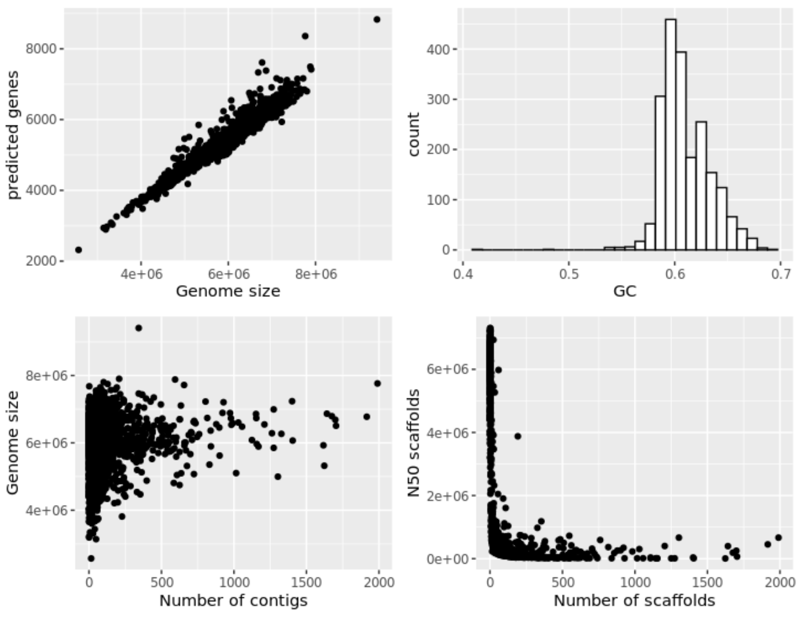

Supplementary Figure 20. Quality report and general statistics of the genomes used in this study. For each genome belonging to *Burkholderia/Paraburkholderia* **a,** and *Pseudomonas* **b,** several parameters were checked: the genome size vs number of predicted genes, histogram of GC% content, number of contigs vs genome size and number of scaffolds vs N50 scaffolds.

Supplementary Figure 21

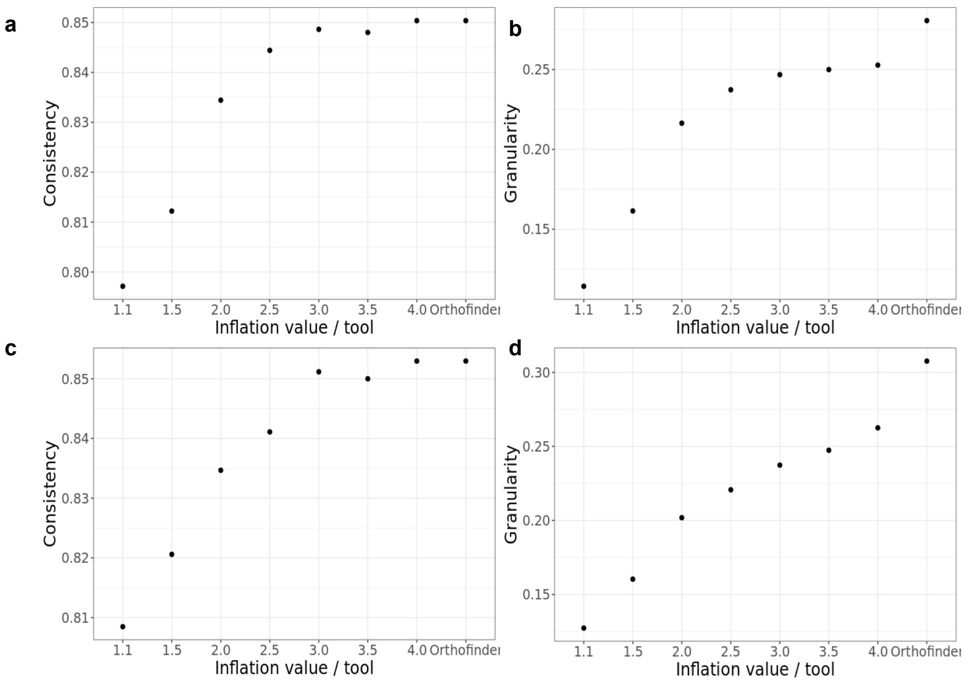

Supplementary Figure 21. Markov Clustering (MCL) inflation value hyperparameter optimization and benchmark with OrthoFinder. Using OrthoFinder[89] as "gold standard" we have evaluated the bacLIFE's gene cluster consistency and granularity among 7 different inflation values in *Pseudomonas* **a, b,** and *Burkholderia/Paraburkholderia* **c, d,** datasets. Consistency is defined as the proportion of gene clusters with all its members being the same EC number (Enzyme Commision number) over the number of gene clusters with at least two members with an EC number. Granularity is defined as the proportion of EC numbers that are in more than one gene cluster.

Supplementary Figure 22

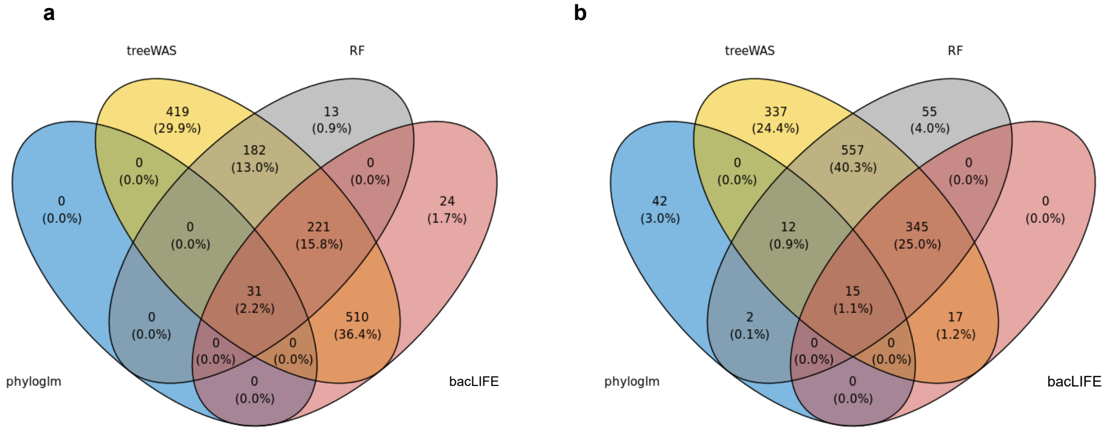

Supplementary Figure 22. Overlap between phyloglm, treeWAS, random forest important variables and bacLIFE LAGs. a and **b,** Venn diagram illustrating the intersection between the LAGs obtained with phyloglm[100] ($p < 0.05$), treeWAS[101] ($p < 0.05$), random forest variables with importance > 0 and the threshold >70% and >2 log2fold change used in this study for *Burkholderia/Paraburkholderia* and *Pseudomonas*, respectively.
