# Supplementary material for: bacLIFE: a user-friendly computational workflow for genome analysis and prediction of lifestyle-associated genes in bacteria
Source: Nat Commun. 2024 Mar 7;15:2072. doi: 10.1038/s41467-024-46302-y (PMC10920822; doi:10.1038/s41467-024-46302-y)
Supplement: Supplementary file 3 — Description of Additional Supplementary Files [file 41467_2024_46302_MOESM3_ESM.pdf]

Description of Additional Supplementary Files:

Supplementary Dataset 1: Input genomes and their clustering at 99 ANI.

Supplementary Dataset 2: Lifestyle metadata before and after applying lifestyle prediction.

Supplementary Dataset 3: p-values resulting from Kruskal-Wallis statistical tests, which were conducted to evaluate the differences among lifestyles within each COG functional category.

Supplementary Dataset 4: Plant pathogen LAGs in *Burkholderia/Paraburkholderia* dataset (Fisher's exact test two-sided, p < 0,01, presence in plant pathogens > 70% and log2fold > 2).

Supplementary Dataset 5: Plant pathogen LAGs in *Pseudomonas* dataset (Fisher's exact test two-sided, p < 0,01, presence in plant pathogens > 70% and log2fold > 2).

Supplementary Dataset 6: Plant pathogen LAGs shared between *Pseudomonas* and *Burkholderia*.

Supplementary Dataset 7: Plant pathogen associated GCFs in *Burkholderia*/*Paraburkholderia* dataset (Fisher's exact test two-sided, presence in plant pathogens > 15% and log2fold > 2).

Supplementary Dataset 8: Plant pathogen associated GCFs in *Pseudomonas* dataset (Fisher's exact test two-sided, p < 0,01 and presence in plant pathogens > 15%).

Supplementary Dataset 9: Input genomes used for hyperparameter tunning of the Markov Clustering.

Supplementary Dataset 10: References used to annotate lifestyle at the species level.

Supplementary Dataset 11: Strains, plasmids and primers used in this study.

Supplementary Dataset 12: List of LAGs selected for mutagenesis.
